# Multilevel fMRI adaptation for spoken word processing in the awake dog brain

**DOI:** 10.1038/s41598-020-68821-6

**Published:** 2020-08-03

**Authors:** Anna Gábor, Márta Gácsi, Dóra Szabó, Ádám Miklósi, Enikő Kubinyi, Attila Andics

**Affiliations:** 1 MTA-ELTE ‘Lendület’ Neuroethology of Communication Research Group, Hungarian Academy of Sciences – Eötvös Loránd University, Pázmány Péter sétány 1/C, 1117 Budapest, Hungary; 2 Department of Ethology, Eötvös Loránd University, Pázmány Péter sétány 1/C, 1117 Budapest, Hungary; 3 MTA-ELTE Comparative Ethology Research Group, Hungarian Academy of Sciences – Eötvös Loránd University, Pázmány Péter sétány 1/C, 1117 Budapest, Hungary

**Keywords:** Neuroscience, Auditory system, Cognitive neuroscience

## Abstract

Human brains process lexical meaning separately from emotional prosody of speech at higher levels of the processing hierarchy. Recently we demonstrated that dog brains can also dissociate lexical and emotional prosodic information in human spoken words. To better understand the neural dynamics of lexical processing in the dog brain, here we used an event-related design, optimized for fMRI adaptation analyses on multiple time scales. We investigated repetition effects in dogs’ neural (BOLD) responses to lexically marked (praise) words and to lexically unmarked (neutral) words, in praising and neutral prosody. We identified temporally and anatomically distinct adaptation patterns. In a subcortical auditory region, we found both short- and long-term fMRI adaptation for emotional prosody, but not for lexical markedness. In multiple cortical auditory regions, we found long-term fMRI adaptation for lexically marked compared to unmarked words. This lexical adaptation showed right-hemisphere bias and was age-modulated in a near-primary auditory region and was independent of prosody in a secondary auditory region. Word representations in dogs’ auditory cortex thus contain more than just the emotional prosody they are typically associated with. These findings demonstrate multilevel fMRI adaptation effects in the dog brain and are consistent with a hierarchical account of spoken word processing.

## Introduction

During spoken word processing, the human brain separates lexical meaning from emotional prosody^1–3^. Lexical processing entails speech sound sequence recognition and the matching of such sequences to previously associated meanings. This requires access to pre-existing speech sound sequence representations, assumedly involving higher levels of the speech processing hierarchy^3,4^. In contrast, emotional prosody processing is largely based upon simple acoustic cues (such as pitch and pitch change)^5–8^. In an fMRI study with awake dogs (*Canis familiaris*) listening to words, we found evidence that the ability to separately process lexical information and emotional prosody is not specific to humans^9^. Dogs showed an overall right hemispheric bias for lexically marked (praise) but not for lexically unmarked (neutral) words, independently of emotional prosody. While this initial study identified a set of auditory brain regions in dogs that are responsive to human speech in general, the distribution of labour among these regions remained unclear. To functionally characterize speech-responsive regions and better understand the relationship of lexical and emotional prosody processing in dogs, here we followed up directly on our previous work, using the same stimuli, but applying a multilevel fMRI adaptation paradigm.

Habituation/dishabituation paradigms are successfully used in various species, including dogs, to examine whether individuals are able to distinguish among certain stimuli^10,11^. This behavioural priming phenomenon is often linked to a reduction in neural activity associated with repeated stimulus processing, which can be measured by single-cell recording^12^, electrophysiological measures^13^ or haemodynamic imaging techniques like PET and fMRI^14^. FMRI adaptation effects (reduction in the BOLD signal after repeated presentations of a stimulus) have been demonstrated in different mammal species (e.g. macaques^15^, rats^16^, and also in humans^17^). FMRI adaptation can occur at different time scales from seconds^18^ to minutes^19,20^. Short-term and long-term fMRI adaptation effects appear to be induced by different underlying mechanisms: short-term or rapid fMRI adaptation^21^ reflects stimulus similarity from the directly preceding stimuli, also referred to as carry-over effects^22^, while long-term fMRI adaptation is thought to have a role in the formation of long-term stored representations and to thus reflect long-lasting neural sharpening for learned stimuli^19,23^. Short-term repetition suppression has also been suggested to reflect initial responses^24^, early sensory, mostly bottom-up processes, while long-term repetition suppression may reflect top-down modulation from regions higher in the processing hierarchy^25,26^. Aging can modulate adaptation effects both neurally and behaviourally. The nature of such modulatory effects is, however, unclear. Age-related differences in fMRI adaptation in humans may be related to the reduction of neural selectivity in older individuals (i.e. neural dedifferentiation^27^), or a decline in inhibitory processes that may result in inefficient filtering of irrelevant stimulus variation^28^.

Auditory fMRI adaptation studies in humans suggest that lexical processing, typically tested by repeated presentations of known words, can be reflected by both long-term^29–31^ and short-term^32,33^ repetition suppression effects. Long-term priming for lexical processing has also been demonstrated behaviourally^30,34,35^. Several areas of the human auditory cortex (e.g. BA 21^36^) and the inferior frontal cortex (e.g. BA 45, 47^36^) are more strongly adapted during lexical than during phonetic tasks, especially in the left hemisphere^30,31,36^. Lexical meaning processing mostly occurs in temporal and frontal areas^7,8,37^. Most human studies on lexical processing reported a clear left hemispheric bias, typically linked to higher levels (mid and anterior STG) of the auditory ventral stream^3,4^.

Emotional prosody processing is highly dependent on acoustic features^37^. After subcortical auditory regions provide a first acoustic analysis of vocal emotions, further integration and cognitive appraisal of the acoustic cues take place in the primary and secondary auditory cortices^6,38,39^. The involvement of both subcortical and cortical auditory regions in processing human emotional vocalizations has also been demonstrated in dogs^40^. Based on both human^37^ and animal experiments ^41,42^, acoustic processing is reflected by adaptation effects already at an early stage of processing, in the subcortical auditory thalamus. In humans, acoustic sensitivity is often shown to be reflected by short-term adaptation effects for consecutive stimuli^43^, however, long-term acoustic adaptation effects over several minutes have also been demonstrated^23,44^.

To dogs, communicating effectively with humans and associating meanings to words is highly relevant^45,46^, but very little is known about the similarities and differences between the auditory mechanisms involved in lexical processing in dogs and humans. Beyond our previous study on dogs’ lexical processing^9^, there have been two recent dog fMRI studies that used words as stimuli, but neither of these two was designed to reveal lexical effects. One study found an increased activity for novel pseudowords compared to trained words in the broadly defined parietotemporal cortex, but that effect was related to novelty processing rather than to lexical processing^47^. The other study showed that stimulus-reward neural associations are formed less effectively for verbal than for visual or olfactory cues^48^. Although fMRI adaptation appears to provide an efficient means to investigate auditory processing mechanisms in a passive listening paradigm, it has never been exploited in dogs before.

In this fMRI experiment, dogs listened to lexically and prosodically marked and unmarked words in all combinations. This way we could separately investigate the effects of lexical and prosodic processing. The term lexically marked (meaningful) word refers to sound sequences that are typically used in the same context: when praising the dog. Lexically unmarked (meaningless) words are not associated with any specific contexts for dogs. We use the term lexical meaning to differentiate it from the intonationally conveyed meaning of a sound sequence—the latter one is reflected in emotional prosody. To avoid speaker-related familiarity and context difference effects, which strongly affect dogs’ behaviour in responses to verbal commands^49^, all words were spoken by a single female trainer, who often talked to all dogs during the several month-long fMRI training process. We used a rapid event-related design, presenting each word as a separate trial and modelling long-term (across the entire run, i.e. 30 repetitions over ~ 6.5 min) and short-term (across consecutive trials, i.e. 3 repetitions within 9 s) repetition effects, to measure fMRI adaptation at different time scales, similarly to previous works^19,23,50^. We hypothesized that in dogs, similarly to humans, lexical and prosodic processing are reflected by distinct fMRI adaptation effects in speech-responsive auditory brain regions, and are modulated by age. More specifically, we predicted that in dogs lexical meaning-based adaptation (1) would be independent of prosody effects at higher levels of the processing hierarchy, and (2) would exhibit right hemisphere dominance.

## Results

**Figure 1:**
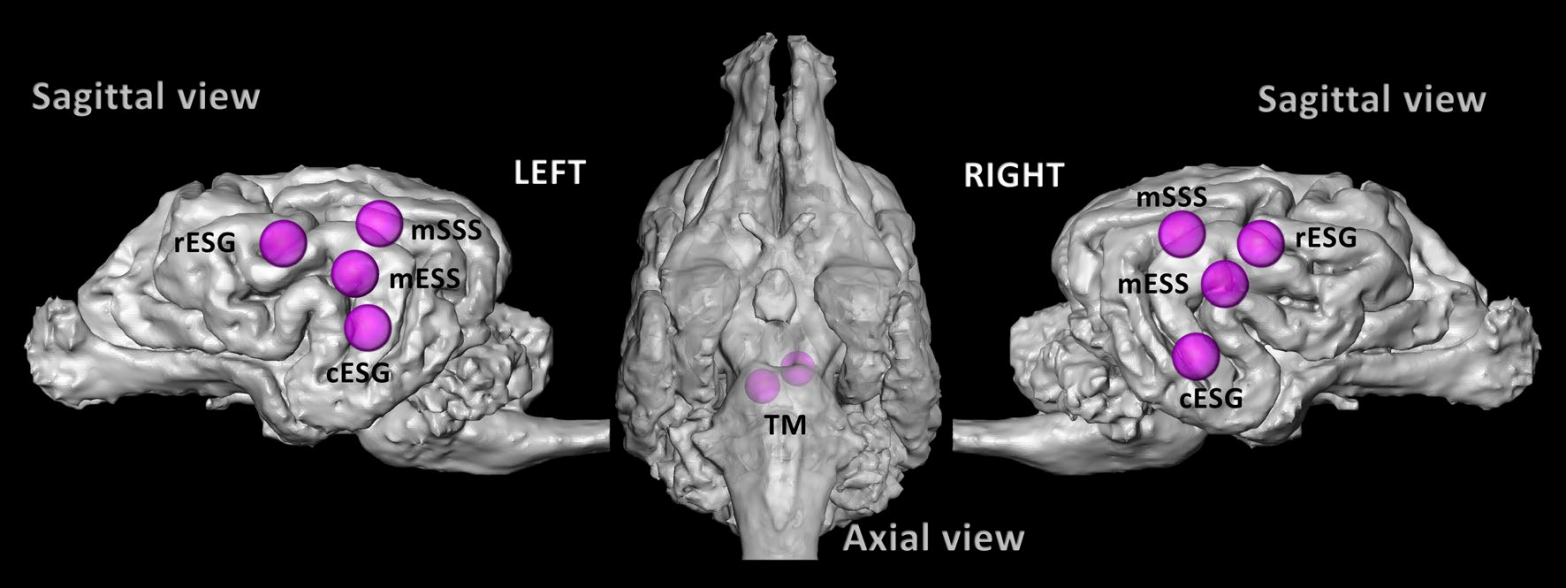
Speech-responsive auditory regions in the dog brain. Purple spheres (R = 4 mm) are centred around previously functionally defined auditory activity peaks (Andics et al., 2016), using a speech vs. silence contrast at the group level with the same dog participants, and used as regional search spaces. Speech-responsive peaks were defined individually within the above spheres (see Supplementary Materials, Table S1), and individual ROIs with 2-mm-radius—used in the later analyses—were created around them. *TM* left tectum mesencephali, *mESS* mid ectosylvian sulcus, *mSSS* mid suprasylvian sulcus, *rESG* rostral ectosylvian gyrus, *cESG* caudal ectosylvian gyrus.

We found no significant effects (voxel-level, FWE-corrected P < 0.05) of either lexical meaning or emotional prosody with the classic 4-condition-based model, neither in whole-brain tests nor in functionally defined speech-responsive regions (Fig. 1).

Next, we performed short- and long-term fMRI adaptation analyses (see Methods for details). Significant main effects and interactions from these analyses are summarized in Table 1.

**Table 1:** FMRI adaptation effects for speech processing in dog auditory regions.

Brain region	Effect	df1	df2	F	P
**Prosody-based short-term fMRI adaptation**					
TM	Repetition	2	22	6.368	0.007
**Lexical meaning-based short-term fMRI adaptation**	–				
**Long-term FMRI (fMRI) adaptation**					
TM	Repetition × prosody × hemisphere	29	290	2.283	< 0.001
mess (mESS)	Repetition × lexical meaning × hemisphere	29	290	1.859	0.006
	Repetition × lexical meaning × prosody	29	290	1.863	0.006
	Repetition × lexical meaning × Age (age)	29	290	2.030	0.002
mSSS	Repetition × lexical meaning × hemisphere	29	290	1.836	0.007
cESG	Repetition × lexical meaning	29	290	2.365	< 0.001

The table lists significant effects of the prosody- and lexical meaning-based short-term fMRI adaptation analyses (RM ANOVAs with factors repetition and hemisphere), and the long-term fMRI adaptation analyses (RM ANOVAs with factors repetition, lexical meaning, prosody, and hemisphere; and covariate age). All analyses were performed in five speech-responsive regions (TM, mSSS, mESS, rESG, cESG), bilaterally (see Fig. 1). No suprathreshold main effects were found. In case of suprathreshold 3-way-interactions, 2-way-interactions with the same factors are not reported (but see text for details on post-hoc tests). Only effects surviving Bonferroni correction for multiple comparisons are reported. N = 12.

**Figure 2:**
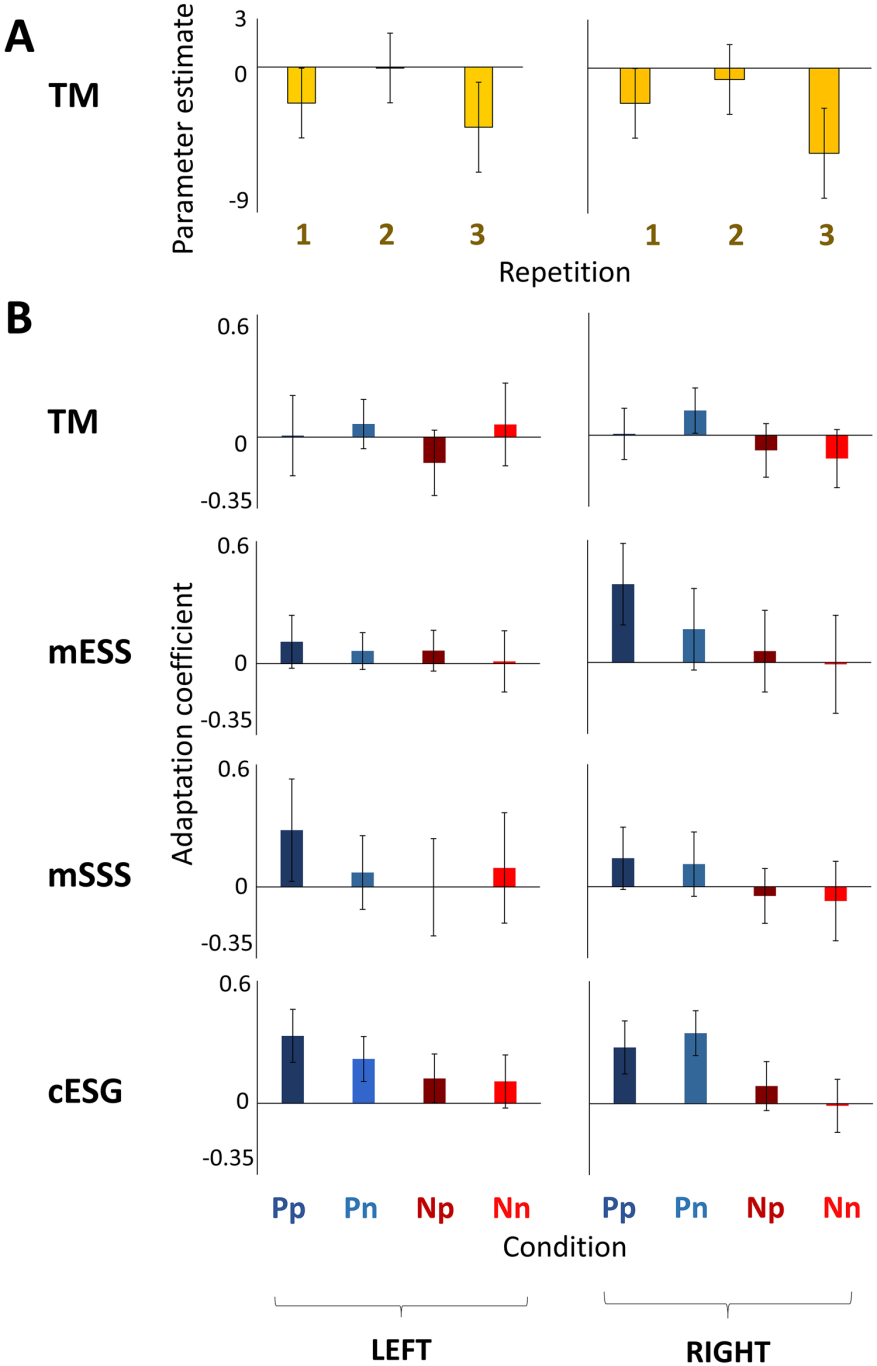
FMRI adaptation effects for speech processing in dogs. (**A**) Prosody-based short-term fMRI adaptation effects. Parameter estimates (trial-based beta values) are averaged for all trials that were the first, second, or third consecutive repetitions of the same prosody. (**B**) Long-term fMRI adaptation effects for prosody and lexical meaning. Adaptation coefficients are defined as the negative of the slope of the linear trendline for trial-based beta values across repetitions (see Methods for details). *Pp* lexically marked (praise) words with praising prosody, *Pn* lexically marked (praise) words with neutral prosody, *Np* lexically unmarked (neutral) words with praising prosody, *Nn* lexically unmarked (neutral) words with neutral prosody. *P < 0.005; **P < 0.001. Error bars represent SEM. N = 12.

The prosody-based short-term fMRI adaptation analyses revealed a bilateral repetition effect in the tectum mesencephali (TM). Follow-up pairwise comparisons in the TM indicated a significant suppression effect between the second and third repetitions (T_11_ = 3.907, P = 0.003), but no difference between the first and second repetitions (T_11_ = − 1.638, P = 0.130) (Fig. 2A). There were no significant repetition or hemisphere effects in any auditory cortical regions. The lexical meaning-based short-term fMRI adaptation analyses revealed no significant effects of either repetition or hemisphere, neither in subcortical nor in cortical speech-responsive regions.

Long-term fMRI adaptation analyses revealed prosody-dependent repetition effects in the subcortical TM and the near-primary auditory cortical mid ectosylvian sulcus (mESS); and lexical meaning-dependent repetition effects in the near-primary mESS and mid suprasylvian sulcus (mSSS), and the secondary auditory cortical caudal ectosylvian gyrus (cESG). Figure 2B displays long-term fMRI adaptation effects. Bar graphs display adaptation coefficient values: the larger the adaptation coefficient, the greater the repetition suppression effect. More specifically, in the subcortical TM, we found a repetition by prosody by hemisphere interaction, indicating repetition enhancement for praising prosody and repetition suppression for neutral prosody, with a larger difference between the two in the left hemisphere. Post-hoc tests for repetition effects in the TM, however, did not reach significance for either praising or neutral prosody, in either hemisphere (Fs < 1.165, Ps > 0.260). In mESS, we found multiple three-way interaction effects, involving the factors repetition, lexical meaning and, as a third factor, hemisphere, prosody or age. Post-hoc tests in the mESS revealed significant repetition suppression for praise words (1) in the right hemisphere (F_29,319_ = 1.954, P = 0.003); (2) in praising prosody (F_29,319_ = 1.779, P = 0.010), and (3) in younger dogs (F_29,145_ = 2.78, P < 0.001). Note that for the post-hoc test age was added as a category variable (young: 2–5 years, N = 6; old: 7–10 years, N = 6, Fig. S1). In mSSS, we found a repetition by lexical meaning by hemisphere interaction, suggesting that lexical meaning-dependent adaptation in this region was stronger for praise words, in the right hemisphere. Post-hoc tests of repetition effects in the mSSS, however, did not reach significance for either praise or neutral words, in either hemisphere (Fs < 1.466, Ps > 0.061). In cESG, we found a repetition by lexical meaning interaction. Post-hoc tests in the cESG revealed significant repetition suppression for praise words (F_29,319_ = 2.179, P = 0.001). No significant main effects or interactions were revealed in the rostral ectosylvian gyrus (rESG).

## Discussion

This study presents the first demonstration of fMRI adaptation effects in the dog brain (but note a recent report of repetition enhancement effects^51^). We used these effects successfully to demonstrate the involvement of certain auditory regions in lexical and prosodic processing. By characterizing neural responses in dogs’ speech-responsive brain regions for auditorily processed words using a multilevel fMRI adaptation paradigm, we found (1) lexical meaning-dependent long-term fMRI adaptation effects in near-primary and secondary auditory cortical regions, and (2) emotional prosody-dependent fMRI adaptation in a subcortical and a near-primary auditory cortical region. Lexical adaptation appeared only cortically and only as a long-term effect. Subcortical auditory regions showed only prosodic but no lexical adaptation. In a near-primary auditory cortical region lexical adaptation showed right-hemisphere bias, was enhanced by emotional prosody, and was modulated by age.

By analysing repetition effects, we demonstrated that three cortical speech-responsive auditory regions (mSSS, mESS, cESG) in dogs are sensitive to the lexical markedness of spoken words: these regions exhibited greater long-term fMRI adaptation—and thus a weaker overall response—for lexically marked (praise) words than for lexically unmarked (neutral) ones. In their studies on humans, Gagnepain et al. (2008)^30^ and Orfanidou et al. (2006)^29^ also found long-term fMRI adaptation (and long-term behavioural priming) for meaningful words in multiple areas of the non-primary auditory cortex (e.g. mSTG, pSTG, L MTG). Additionally, Gold et al. (2005)^36^ found stronger long-term fMRI adaptation during a lexical meaning-related task than during a phonological task in the left middle temporal gyrus (L MTG) and the left inferior frontal gyrus (L IFG). In addition, we found no short-term lexical adaptation effects. Previous literature is inconclusive on whether in humans lexical processing is reflected also in short-term^33^, or only in long-term adaptation effects^29,30^. Nevertheless, short-term repetition effects are usually reported for the repetitions of simple, stimulus-dependent cues (such as emotional prosody cues), rather than for abstract stimulus properties (such as lexical meaning)^19,24–26^. Our findings thus suggest that in dogs, as in humans^29,30,52^, lexical processing is reflected in fMRI adaptation effects in a longer time scale in higher-level cortical regions.

The right hemisphere bias for lexical adaptation in the near-primary auditory cortex corroborates our earlier results^9^, suggesting that the processing of lexically marked words in dogs is more pronounced in the right hemisphere. Across auditory cortical regions, we found hemispheric bias only for lexical but not for prosodic markedness. In humans, lexical meaning processing shows hemispheric asymmetry towards the left hemisphere of the brain^2,4^, while highly emotional speech stimuli are processed with lateral symmetry^5^ or with a right bias^5,53^. In dogs, behavioural measures were used in two recent studies to search for the possible presence of functional hemispheric asymmetries for processing human speech. While no consistent head-turn bias was found for naturally spoken meaningful instruction words in either study^54,55^, right head-turn bias (possibly indicating left hemispheric bias) was found for commands where meaningful phonemic cues were made salient artificially, and left head-turn bias (perhaps indicating a right hemispheric bias) for commands where emotional prosodic or speaker-related cues were made salient artificially^54^. While the present fMRI findings corroborate earlier neuroimaging results^9^, it is harder to reconcile them with behavioural reports. One possibility is that behavioural measures of lateralization do not reflect functional hemispheric asymmetries as directly as it has often been proposed. A combined behavioural-fMRI lateralization study in humans also demonstrated that orienting biases for speech stimuli are not necessarily coupled with lateralized processing^56^. Combined behavioural-fMRI investigations would have the capacity to reveal the neural pattern behind orienting biases. Another possible explanation for the seemingly contradicting findings is that the right bias for meaningful words presented here reflected the recognition of the processed lexical item (i.e. access to learned speech sound sequences), while the right head-turn bias in the behavioural study^54^ may have revealed a left bias for segmental analysis (i.e. identifying phonemes in a speech stream), a necessary prerequisite of lexical processing. Left bias for segmental and right bias for suprasegmental processing is consistent with an acoustic account of lateralization (i.e. short vs long temporal windows for processing in left vs right auditory cortex, respectively)^37,57^. We suggest that this account can explain many of the findings of Ratcliffe and Reby’s (2014)^54^ study. In our study, neither segmental nor speaker-related suprasegmental cues have been varied systematically, and prosodic suprasegmental cues did not lead to a hemispheric bias in the auditory cortex (and in the subcortical TM, the only region with hemisphere bias for prosodic adaptation, we found that prosodically more salient stimuli did not elicit stronger adaptation than neutral prosody in either hemisphere), so our findings neither support nor contradict the assumptions of the acoustic account of lateralization. Instead, our findings support a functional, meaningfulness-based account of lateralization. Hemispheric effects for processing meaningful, relevant sounds have been found in many species, including birds, non-primate mammals, and primates^58–61^, even though most of these showed a left bias (but see^62^). Note however that most of these studies tested conspecific sounds. It is possible that the recognition of a learned auditory stimulus elicits hemispheric bias in dogs, and while this bias is typically left-sided for conspecific vocal sounds, it becomes right-sided for vocalizations that elicit intense emotions (cf.^55,60,63^).

Next to lexical adaptation, this study also showed evidence for emotional prosodic adaptation effects in the dog brain. We found short-term and long-term prosodic adaptation effects in a subcortical auditory region (TM), and long-term prosodic adaptation effect in a near-primary cortical auditory region (mESS). The involvement of the subcortical TM reflects the role of these early-stage areas in processing acoustic cues relevant to emotional prosody. According to single-unit experiments, in many species, the subcortical auditory thalamus shows short-term adaptation to stimulus repetitions^41,42^. Anatomically, the speech-responsive TM region we used here involves the dog auditory thalamus, but the spatial resolution of the present study does not allow for its disentanglement from other, neighbouring subcortical structures. FMRI evidence for the involvement of early subcortical levels of the auditory pathway for processing vocal sounds has been reported for both humans^37^ and dogs^40^. The other prosody-sensitive region, mESS is centred around the sulcus located at the border of the mid ectosylvian gyrus (mESG), the primary auditory cortex of the dog, a region that receives tonotopic input from the auditory thalamus^64^. We found that mESS is the single cortical speech-responsive region where long-term fMRI adaptation was dependent not only on lexical meaning but also on prosody (being strongest for praise words in praising prosody). These findings suggest that the analysis of emotional prosody information in speech involves early levels of the auditory processing hierarchy in dogs.

Our findings suggest that dogs, similarly to humans, process emotional prosodic cues in spoken words at lower levels (subcortical and near-primary cortical regions, reflected in both short-term and long-term adaptation effects) and lexical information at higher levels (near-primary and secondary auditory cortical regions, reflected in long-term adaptation effects) of the auditory processing hierarchy. Prosody processing was thus subcortically independent of lexical cues, prosody influenced lexical processing in a near-primary cortical region and, finally, lexical processing was independent of prosodic cues in a secondary auditory cortical region. This hierarchical organization may reflect similarities of dog and human speech processing, but this does not imply that this processing hierarchy is of linguistic nature. Indeed, the prosodic-lexical hierarchy reported here and also in humans may reflect a more general, not speech-specific processing principle. According to Pessoa and Adolphs (2010)^65^, perceptually salient (e.g. emotionally loaded, motivationally important) cues are typically analysed at lower levels (“low road”), and more complex, learnt, perceptually less salient cues of the same signal are analysed at higher levels (“high road”). This low road / high road processing hierarchy has been demonstrated in multiple species, independently of a linguistic context^66–68^. In the present study, the prosodic manipulation was acoustically salient, as praising prosody was characterized by a higher pitch and pitch range than neutral prosody. In contrast, lexically marked and unmarked stimuli did not systematically differ in acoustic cues (contrasted conditions were matched for consonant–vowel structure and for emotional prosody), this learnt distinction was not salient acoustically. Note that there were also no familiarity differences between lexically marked and unmarked words because the lexically unmarked (neutral) words we selected here were words that had been used with a similar frequency to praise words in everyday speech, so the actual sound sequences were similarly familiar to dogs. Therefore, the only systematic difference between praise words and neutral words was that praise words were arbitrary sound sequences with an associated meaning, while neutral words were arbitrary sound sequences with no associated meaning. This contrast between emotional prosodic and lexical cues is not specific to our study—instead, it is a basic, essential difference between prosodic and lexical information and also applies to speech processing in humans.

So, does the reported lexical effect constitute evidence for human-analogue lexical representations in the dog brain? We do not suggest that the neural speech processing hierarchy shown here reflects any linguistic capacity in dogs. In contrast, our findings indicate that some of the neural mechanisms that support lexical processing may not be specific to humans. The reported lexical effect in dogs reveals differential processing of meaningful and meaningless words. Importantly, the fact that the presence of an associated meaning made a difference to the processing of a sound sequence in dogs, does not reveal lexical access. In other words, we do not know whether dogs learnt the lexical meaning (i.e. praise) associated to certain sound sequences (i.e. the praise words), or this association simply made those sound sequences more relevant to them and therefore easier to learn (and then recognize as known sound sequences). Consequently, we do not propose that dogs have human-analogue lexical representations, or that the observed lexical effects reveal complex or abstract processes. The only level of abstraction we argue for is regarding acoustics: although usually dogs only hear praise words in praising prosody, the lexical effect for praise words in secondary auditory regions was not stronger for praising than neutral prosody, suggesting that word representations in dogs’ auditory cortex thus contain more than just the emotional prosody they are typically associated with. The neural mechanisms underlying the reported lexical effects may not involve both core components of lexical processing (i.e. sound sequence recognition and meaning extraction). Based on this study alone we cannot claim that praise words, unlike neutral words, have been meaningful to dogs, it is also possible that the corresponding sound sequences were simply better learnt. Future studies will need to determine whether the neural process underlying this lexical effect reflects sound sequence recognition or word meaning extraction. Both accounts are plausible. Comparative behavioural work demonstrated that the dissociation of sound sequence and pitch during auditory processing is not unique to humans (e.g. dolphins^69^, songbirds^70^). But there is also evidence that at least some dogs can associate meaning to words (see^71,72^ for case studies with dogs correctly identifying hundreds of toys based on their name). Either way, the effect we report here evidences learning about words in dogs and cannot be accounted for by differences in acoustics or frequency-based familiarity.

We found no lexical or prosodic effects in a standard GLM-based analysis: this test revealed no brain region in dogs in which praise words or praising prosody elicited stronger or weaker overall activity than neutral words or prosody. This negative finding is not surprising, as the event-related design applied here, while more suitable to investigate across-trial dynamics of brain responses, is known to be less robust to overall condition differences^73^. Furthermore, the same contrasts did not show strong effects in speech-responsive auditory regions in a previous, block-design study either^9^. This shows that direct comparisons do not always constitute the optimal analysis of condition differences in fMRI: in the present case, an adaptation analysis was more informative.

The present study also showed that age modulates lexical adaptation effects in dogs. Specifically, the mESS adaptation effect difference between known and unknown words was larger in younger dogs. The small sample size of the present study does not allow for any strong conclusions on between-subject factors such as age. Nevertheless, it is worth noting that in humans, age effects have also been more pronounced in abstract, lexical/semantic components of repetition priming for language processing than in primary, perceptual components^74^. Also, the reduced fMRI adaptation difference between conditions in older individuals supports the account that neural specificity decreases with age^27,75^.

One limitation of the present study is that all stimuli were recorded from a single speaker, a female trainer of all tested dogs. While this might make our results less generalizable, we decided on using a single speaker with consideration to the reports that dogs process human vocal sounds in a highly context-sensitive manner^76^, and that speaker familiarity affects their behavioural responses to instruction words^49^. We aimed at using identical stimuli across participants and also maximizing the relevance of our stimuli in this sense, similarly to other studies using a trainer’s voice instead of a set of less familiar speakers (cf.^71^). Human fMRI studies on speech processing also often use a single speaker^77,78^. One could argue that overall adaptation effects for speech stimuli may be different for unfamiliar speakers^79^. Crucially, however, the adaptation effects here were all condition-dependent, that is, stimuli in one condition elicited stronger adaptation than stimuli in another condition, even though all were spoken by the same speaker. We cannot draw conclusions about the across-speaker generalizability of lexical representations in dogs based on the present study, but this does not question the lexical nature of the lexical adaptation effects we demonstrated here. Another limitation is that the lexically marked words we chose were all used in a single context: when rewarding the dog. Therefore, based on this study alone we cannot determine whether the revealed right bias (and other lexical effects) reflects lexical processing (sound sequence recognition or meaning extraction) in general or, more specifically, the processing of praise words spoken by a familiar person. To better understand the mechanisms of spoken word processing in dogs, further fMRI studies are required. In these future studies it will be important to test the role of speaker familiarity and the processing of words learned in different contexts (e.g. applying object names or instructions). Widening the framework in which dogs’ neural responses to human vocal/verbal communication is investigated would be intriguing because the dog has been recently suggested as a complementary model species to the traditionally used primate and rodent models due to its evolutionary and ontogenetic development in the human social environment and also for many practical reasons (e.g., non-invasive measurements, ethical issues)^46,80–82^.

This study demonstrated the usefulness of a multilevel fMRI adaptation approach to functionally characterize speech-responsive regions in the dog brain. We identified speech-responsive auditory regions involved in lexical and emotional prosody processing in dogs. We replicated our earlier findings^9^ that in dogs, lexically marked praise words are processed with a right-hemisphere bias. Lexical and prosodic adaptation patterns differed both temporally (long-term effects mostly for lexical processing and short-term effects only for emotional prosody processing) and spatially (lexical processing only cortically, in near-primary and secondary speech-responsive auditory regions, and emotional prosody processing only in subcortical and near-primary auditory cortical regions), suggesting that they indeed reflected distinct stages of an auditory processing hierarchy in the dog brain. Our findings thus provide evidence for the hierarchical processing of spoken words in a speechless species.

## Materials and methods

### Participants

We tested 12 pet dogs (mean age (year) ± SD 6.17 ± 2.82, range 2–10 years; 3 breeds: 6 border collies, 5 golden retrievers, 1 German shepherd; 8 males and 4 females) living in human families. As this work is a follow-up on a previous study^9^, we used the same dog participants (all but one dog participated in the previous study; two dogs that participated in the previous study were not available for measurements any more). The training procedure for dogs to lie motionless throughout the test was based on individual and social learning using positive reinforcement and has been described in detail previously^40^.

### Stimuli

The stimuli were lexically marked (praise) words, meaningful for the dogs, and lexically unmarked (neutral) words, meaningless for the dogs, with praising and neutral prosody in all combinations, identical to those used in Andics et al. (2016)^9^. The three lexically marked (praise) words in Hungarian were: azaz [‘ɒzɒz] / ügyes [‘yɟɛʃ ] / jól van [‘joːlvɒn] for "that's it / clever / well done", all used to praise the tested dogs. As lexically unmarked (neutral) words, we used three conjunction words: akár [‘ɒkaːɾ] / olyan [‘ojɒn] / mégsem [‘meːgʃɛm] for "as if / such / yet", used with similar frequency in everyday speech, but not used in dog-directed speech. We recorded all six words, both with praise and neutral prosody twice (24 recordings in total). A female trainer of the dogs (MG) spoke the words, and she was always present at the scanner during the test sessions. The praising prosody stimuli were characterized by higher pitch and greater pitch range than the neutral prosody stimuli (praising / neutral prosody: mean (F0) = 268(± 20)/165(± 6)Hz, F_1,20_ = 289.725, P < 0.001; mean(F0 range) = 277(± 93)/46(± 9)Hz, F_1,20_ = 68.264, P < 0.001), independently of lexical markedness. There were no systematic pitch or pitch range differences between lexically marked (praise) words and lexically unmarked (neutral) words. To ensure that the stimulus voice has typical acoustic variation, we recorded the same praise and neutral words with both prosodies from 14 other persons and compared the pitch parameters of these reference voices across conditions via RM ANOVA. Here, again, words with praising prosody had higher pitch and higher pitch range than words with neutral prosody (praising / neutral prosody: mean(F0) = 216(± 67)/161(± 55)Hz, F_1,13_ = 67.122, P < 0.001; mean(F0 range) = 144(± 71)/37(± 18)Hz, F_1,13_ = 44.032, P < 0.001), but we found no systematic acoustic differences between praise and neutral words (all Fs < 1).

### FMRI experimental design

We used four speech conditions (with three words per condition): (1) lexically marked (praise) words with praising prosody (Pp), (2) lexically marked (praise) words with neutral prosody (Pn), (3) lexically unmarked (neutral) words with praising prosody (Np), and (4) lexically unmarked (neutral) words with neutral prosody (Nn). We also added a silent condition and used it as a baseline in later analyses. A semi-continuous event-related fMRI paradigm was applied, in which each stimulus was played in 1 s long silent gaps (one stimulus per gap) between 2 s long volume acquisitions. Stimulus onsets were at 0.05 s within the silent gaps. Word lengths were between 0.484–0.896 s (0.642 s on average). One measurement consisted of 135 stimulus presentations (30 of each main condition, with 10 repetitions of every single word with both prosodic patterns, and 15 silent events). A semi-random stimulus order was used, with the proviso that two consecutive stimuli are not the same words with the same prosody. Conditions were evenly distributed, but the order was otherwise random and varied across participants. The experiment consisted of a single approximately 6.5-min run for each dog (the total duration of the run is limited by how long dogs can be instructed to lay motionless).

### Scanning procedure

During scanning, the stimulus presentation was controlled by MatLab (version 9.1) Psychophysics Toolbox 3^83^ and synchronized with volume acquisitions by TTL trigger pulses. Stimuli were presented via MRI-compatible sound-attenuating headphones (MR Confon) that also protected the ears of the dogs from scanner noises. A Philips 3 T whole-body scanner and a Philips SENSE Flex Medium coil were used to perform the measurements, at the MR Research Centre of the Semmelweis University, Budapest. For functional scans, we used a single-shot gradient-echo planar imaging (EPI) sequence to acquire volumes of 29 transverse slices, with 0.5 mm gaps, covering the whole brain (slice order: ascending; spatial resolution including slice gaps: 3.5 × 3.5 × 3.5 mm; TR: 3.0 s; TE: 36 ms; flip angle: 90°; 64 × 64 matrix). One measurement consisted of 139 volumes. A T1-weighted anatomical brain image was acquired in a separate session (turbo-field echo (TFE) sequence; spatial resolution: 1 × 1 × 1 mm, 180 slices).

Our subjects had previously been trained to lie motionless for ~ 8 min without any restriction. We applied an absolute head motion threshold of 2 mm (for each translation direction) and 2° (for each rotation direction) across the entire run. To search for possible condition effects on head motion, we calculated framewise displacement (FD) in each dog (mean FD = 0.23(± 0.15) mm)^84–86^. This average FD value is comparable to a typical human adult’s movement parameters measured in event-related task fMRI studies^87^. Head motions following sound and silence conditions did not differ (T_14_ = − 0.836, p = 0.417). RM ANOVA on dogs’ FD values revealed no systematic differences in head motion across acoustic conditions (lexical meaning: F_1,29_ = 0.009, p = 0.926; prosody: F_1,29_ = 2.129, p = 0.155; lexical meaning × prosody: F_1,29_ = 0.050, p = 0.825).

### FMRI data coding and statistical analysis

FMRI data preprocessing and analysis were performed using the SPM8 toolbox (www.fil.ion.ucl.ac.uk/spm) of MATLAB R2013a (https://www.mathworks.com/products/matlab/). Preprocessing procedure was identical to that in^9^ and involved manual and automatic spatial realignment, coregistration, normalization to an anatomical template, and smoothing. Individual statistical maps were obtained based on the general linear model. We specified two models. For a standard analysis with condition-based contrasts, we modelled the 5 main conditions (Pp, Pn, Np, Nn, and Sil) and used condition regressors. In a second GLM for the fMRI adaptation analyses, each trial (4 speech conditions × 30 repetitions) was modelled separately, and we used trial-based regressors.

To individuate the functional localization of speech-responsive auditory brain areas, we defined with the help of a previous study^9^ with the same stimuli and participants. There, group-level activity peaks of the speech (all conditions) vs silence contrast included the following bilateral auditory subcortical and cortical regions: left and right tectum mesencephali (L TM: − 4, − , − 12 R TM: 2, − 12, − 10), mid suprasylvian sulcus (L mSSS: − 16, − 14, 16; R mSSS: 18, − 14, 14), mid ectosylvian sulcus (L mESS: − 28, − 10, 8; R mESS: 22, − 6, 6), rostral ectosylvian gyrus (L rESG: − 22, 2, 14; R rESG: 20, − 2, 14), caudal ectosylvian gyrus (L cESG: − 24, − 10, − 2; R cESG: 26, − 10, − 6) (Fig. 1). These coordinates (mm) denote left to right, posterior to anterior, and inferior to superior directions respectively, using the same dog brain template space as in Andics et al. (2016)^9^. We created spheres (r = 4 mm) around these peaks and used them as regional search spaces. We then determined the speech-responsive peak within each of these regional search spaces at the individual level (for a list of individual coordinates, see Table S1). In case of a single dog (D12) who did not participate in the previous study, we used group-level peaks. We created spherical ROIs around these individually specified peaks (r = 2 mm). Therefore, each dog had a unique set of ROIs, which were nevertheless determined within the group analysis-based regional search spaces. A similar method was used in a recent dog fMRI study by our group, see^51^. Using trial-based regressors, we then determined parameter estimates (beta values) for each event, averaged within the above described, individual ROIs using WFU PickAtlas^88^.

To investigate long-term condition-dependent fMRI adaptation, we coded each event with reference to the number of preceding repetitions (1 to 30) of the same condition within the test run (e.g. Pp1, Pp2, Pp3…. Pp30) (Table 2). We then compared event-specific parameter estimates (beta values) using RM ANOVAs with hemisphere (left, right), lexical meaning (P, N), prosody (p, n) and repetition (1, 2 … 30) as within-subject factors and age as a covariant within each speech-responsive region. We applied Bonferroni correction for the number of ANOVAs performed (i.e. the number of bilateral ROIs). If a significant interaction with repetition was found, we carried out follow-up tests to investigate repetition effects for each level of the other contributing factors. To illustrate long-term fMRI adaptation effects, adaptation coefficient values were calculated (Fig. 2B): first, we fitted a linear trendline to parameter estimates across repetitions, for each condition; second, we calculated the slope of this trendline, i.e., the rate of BOLD response decrease; third, we took the negative of this slope. Correspondingly, the larger the adaptation coefficient value, the greater the fMRI adaptation (repetition suppression) effect. In an additional analysis, we performed another series of RM ANOVAs to search for possible short-term fMRI adaptation effects for lexical meaning and prosody. For this, we separately coded trials for lexical meaning (P included Pp and Pn, N included Np and Nn) and prosody (p included Pp and Np, n included Pn and Nn). To investigate short-term lexical meaning-based fMRI adaptation we coded every trial based on lexical meaning (P, N) and repetition, i.e. the number of directly preceding trials with the same lexical meaning (1, 2, 3). For example, P2 referred to a praise word that was the second consecutive repetition of the same lexical meaning. We included events until up to 3 repetitions, as 4 or more consecutive repetitions of the same lexical meaning were rare (2.5% of all cases).

**Table 2:** Illustration of coding condition-dependent long-term repetitions, and prosody-based and lexical meaning-based short-term repetitions.

Stimulus order	Pp	Pn	Pp	Sil	Nn	Pn	Nn	Np	Sil	Np	Nn	…	Pp
Prosody-based STA:	p1	n1	p1	–	n1	n2	n3	p1	–	p1	n1	…	p1
Lexical meaning-based STA:	P1	P2	P3	–	N1	P1	N1	N2	–	N1	N2	…	P1
Condition-dependent LTA:	Pp1	Pn1	Pp2	–	Nn1	Pn2	Nn2	Np1	–	Np2	Nn3	…	Pp30

*STA* short-term fMRI adaptation; *LTA* long-term fMRI adaptation; *P* lexically marked (praise) words; *N* lexically unmarked (neutral) words; *p* praising prosody; *n* neutral prosody; *Sil* silence.

To investigate short-term prosody-based fMRI adaptation we coded events similarly, but now based on prosody (p, n) and repetition (1, 2, 3) (Table 2). We then applied RM ANOVAs on beta values for each speech-responsive region with the factors hemisphere and repetition. Only effects that survive Bonferroni correction are reported.

### Ethical statement

Research was done in accordance with the Hungarian regulations on animal experimentation and the Guidelines for the use of animals in research described by the Association for the Study Animal Behaviour (ASAB). Ethical approval was obtained from the local ethical committee (Állatkísérleti Tudományos Etikai Tanács KA-1719 / PEI/001/1,490–4/2015, Budapest, Hungary; Pest Megyei Kormányhivatal Élelmiszerlánc-Biztonsági és Állategészségügyi Igazgatósága XIV-I-001/520–4/2012, Budapest, Hungary). The dog owners were volunteers who received no monetary compensation and gave their written consent to participate with their dogs in the study.

## Supplementary information

Supplementary file1 (PDF 208 kb)
