# Supplementary material for: Multilevel fMRI adaptation for spoken word processing in the awake dog brain
Source: Sci Rep. 2020 Aug 3;10:11968. doi: 10.1038/s41598-020-68821-6 (PMC7398925; doi:10.1038/s41598-020-68821-6)
Supplement: Supplementary file 1 — Supplementary file1 (PDF 208 kb) [file 41598_2020_68821_MOESM1_ESM.pdf]

Supplementary Information

Multilevel fMRI adaptation for spoken word processing in the awake dog brain

Anna Gábor[1*], Márta Gácsi[2,3], Dóra Szabó[2], Ádám Miklósi[2,3], Enikő Kubinyi[2], Attila Andics[1,2]

[1]MTA-ELTE 'Lendület' Neuroethology of Communication Research Group, Hungarian Academy of Sciences – Eötvös Loránd University, H-1117 Budapest, Pázmány Péter sétány 1/C, Hungary; +36-1-381-2179.
[2]Department of Ethology, Eötvös Loránd University, H-1117 Budapest, Pázmány Péter sétány 1/C, Hungary; +36-1-381-2179.
[3]MTA-ELTE Comparative Ethology Research Group, Hungarian Academy of Sciences – Eötvös Loránd University, H-1117 Budapest, Pázmány Péter sétány 1/C, Hungary; +36-1-381-2179.
*Correspondence: annagabor33@gmail.com

Fig. S1. Age-dependent long-term fMRI adaptation effects.
Young represents dogs between age 2-5 years (N = 6), Old represents dogs between age 7-10 years (N = 6). Note that during the main analysis age was used as a continuous variable. P: lexically marked (praise) words; N: lexically unmarked (neutral) words. Error bars represent SEM.

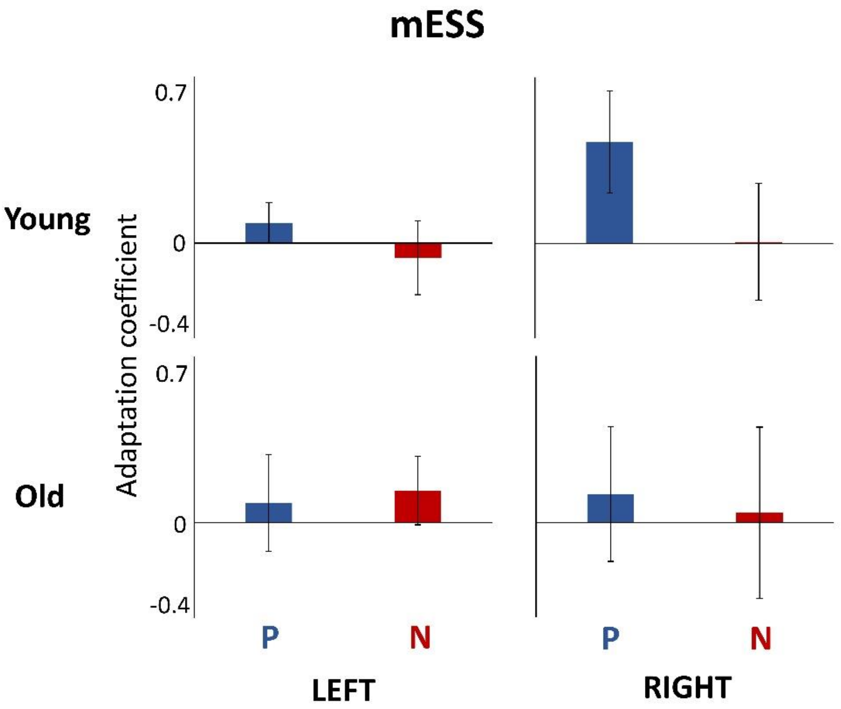

Table S1. Peaks of individually defined ROIs.

**Brain Region**	**Dog**	**Coordinates**					
		**Left**			**Right**		
		**X**	**Y**	**Z**	**X**	**Y**	**Z**
TM	D01	-2	-10	-12	4	-12	-12
	D02	-6	-8	-10	4	-10	-10
	D03	-4	-8	-12	0	-10	-10
	D04	-4	-10	-14	2	-10	-10
	D05	-2	-10	-12	0	-10	-10
	D06	-2	-10	-12	4	-12	-12
	D07	-2	-8	-10	0	-10	-10
	D08	-6	-8	-14	4	-10	-10
	D09	-2	-8	-14	2	-10	-12
	D10	-4	-6	-14	2	-12	-12
	D11	-6	-10	-12	4	-12	-12
	D12	-4	-8	-12	2	-12	-10
mESS	D01	-26	-10	6	22	-10	6
	D02	-24	-10	8	22	-8	4
	D03	-28	-10	10	22	-10	6
	D04	-26	-10	8	22	-8	6
	D05	-26	-8	10	24	-4	4
	D06	-24	-10	8	22	-10	6
	D07	-28	-10	12	20	-8	6
	D08	-26	-8	10	24	-6	4
	D09	-26	-8	6	22	-8	4
	D10	-26	-8	10	24	-4	8
	D11	-26	-12	6	22	-6	10
	D12	-28	-10	8	22	-6	6
mSSS	D01	-20	-14	16	22	-14	14
	D02	-16	-14	12	18	-14	10
	D03	-14	-12	14	18	-10	14
	D04	-20	-14	16	16	-16	12
	D05	-12	-14	16	16	-12	16
	D06	-18	-12	14	22	-14	14
	D07	-16	-14	20	16	-12	12
	D08	-18	-12	18	20	-12	12
	D09	-18	-12	16	20	-16	14
	D10	-14	-12	18	20	-12	12
	D11	-18	-12	14	18	-12	12
	D12	-16	-14	16	18	-14	14
rESG	D01	-20	0	12	24	-2	14
	D02	-22	-2	14	22	-4	12
	D03	-22	-2	14	20	-6	14
	D04	-20	2	12	18	0	12
	D05	-24	4	12	20	-6	14
	D06	-22	-2	14	20	-6	14
	D07	-22	2	18	22	0	12
	D08	-22	-2	14	22	-4	14
	D09	-22	-2	14	22	-4	12
	D10	-18	2	14	18	0	14
	D11	-24	0	16	18	-4	16
	D12	-22	2	14	20	-2	14

cESG	D01	-22	-10	0	26	-12	-6
	D02	-24	-10	-6	24	-12	-8
	D03	-26	-8	0	26	-10	-10
	D04	-24	-6	-2	24	-12	-6
	D05	-22	-8	0	28	-12	-6
	D06	-22	-8	0	26	-10	-2
	D07	-24	-10	-6	28	-8	-6
	D08	-26	-8	0	26	-6	-6
	D09	-24	-6	-2	26	-6	-6
	D10	-22	-8	0	24	-10	-4
	D11	-22	-12	-2	24	-8	-8
	D12	-24	-10	-2	26	-10	-6

The table lists individual speech-responsive peaks per region. Regional search spaces (r = 4 mm) were centred around independently, functionally defined speech-responsive peaks, using a speech vs. silence group level contrast with the same dog participants[1]. Within the regional search spaces, individual speech-responsive peaks (shown in this table) were defined based on the same (independent from the current) dataset. In case of a single dog (D12) who did not participate in the previous study, we used group-level peaks. Spherical ROIs (r = 2 mm) were created around these individual peaks, and used in all analyses. Coordinates (mm) denote left to right, posterior to anterior, and inferior to superior directions respectively, using the same dog brain template space as in Andics et al. (2016)[1].

References

Andics, A. et al. Neural mechanisms for lexical processing in dogs. Science, 353, 1030–1032 (2016).
